# Retraction Note: Nucleic acid sensing activates the innate cytosolic surveillance pathway and promotes parasite survival in visceral leishmaniasis

**DOI:** 10.1038/s41598-022-09098-9

**Published:** 2022-03-22

**Authors:** Sushmita Das, Ashish Kumar, Abhishek Mandal, Kumar Abhishek, Sudha Verma, Ajay Kumar, Pradeep Das

**Affiliations:** 1 Department of Microbiology, All-India Institute of Medical Sciences (AIIMS), Patna, India; 2 Department of Biochemistry, ICMR-Rajendra Memorial Research Institute of Medical Sciences, Patna, India; 3 Department of Molecular Biology, ICMR-Rajendra Memorial Research Institute of Medical Sciences, Patna, India

Retraction of: *Scientific Reports* 10.1038/s41598-019-45800-0, published online 08 July 2019

The Editors have retracted this Article.

After publication, concerns were raised regarding a number of figures in the Article. Specifically:

- Western blot images presented in Figures 1E and 2C appear to show the same result;
- in Figure 3G, the DAPI panels appear to originate from the same image, as the Mock, Ld-DNA treated and Ld-DNA+DOTAP panels all appear to partially overlap with the Ld-DNA+Lipo2000 panel (rotated);
- the DAPI Mock and Ld-DNA+DOTAP panels appear to contain some duplicated cells;
- the p-TBK1 Mock, Ld-DNA treated and Ld-DNA+DOTAP images do not appear to contain any signal;
- Figure 5F appears to contain inconsistencies in the background of the tubulin bands.

The Authors were not able to provide the original data requested by the Editors. The Editors therefore no longer have confidence in the presented data.

Pradeep Das and Kumar Abhishek disagree with this retraction. None of the other Authors responded to correspondence from the Editors about this retraction.

